# A novel role for microRNA-129-5p in inhibiting ovarian cancer cell proliferation and survival via direct suppression of transcriptional co-activators YAP and TAZ

**DOI:** 10.18632/oncotarget.3254

**Published:** 2015-04-07

**Authors:** Guosheng Tan, Xinping Cao, Qiangsheng Dai, Bing Zhang, Jianwen Huang, Shiqiu Xiong, Yong yu Zhang, Wei Chen, Jianyong Yang, Heping Li

**Affiliations:** ^1^ Department of Interventional Radiology, the First Affiliated Hospital of Sun Yat-sen University, Guangzhou, China; ^2^ Department of Radiotherapy, Sun Yat-sen University Cancer Center, Guangzhou, China; ^3^ Department of Medical Oncology, the First Affiliated Hospital of Sun Yat-sen University, Guangzhou, China; ^4^ Department of Nuclear Medicine, the First Affiliated Hospital of Sun Yat-sen University, Guangzhou, China; ^5^ Department of Radiation Oncology, the First Affiliated Hospital of Sun Yat-sen University, Guangzhou, China; ^6^ Department of Biochemistry, University of Leicester, Leicester, UK

**Keywords:** miR-129-5p, YAP, TAZ, cell proliferation, survival

## Abstract

Transcriptional co-activator Yes-associated protein (YAP) and transcriptional co-activator with PDZ-binding motif (TAZ) are key oncogenes in mammalian cells. Activities of YAP and TAZ are largely restricted by the Hippo tumor suppressor pathway through phosphorylation-ubiquitination mechanisms. The involvement of microRNA in cancer progression has recently been reported, though whether they have a role in activating YAP and TAZ in human cancer cells remains unclear. Here, we report a microRNA, miR-129-5p, directly represses YAP and TAZ expression, leading to the inactivation of TEA domain (TEAD) transcription, and the downregulation of Hippo downstream genes, connective tissue growth factor (CTGF) and Cyclin A. Furthermore, we reveal miR-129-5p inhibits ovarian cancer cell proliferation, survival and tumorigenicity, and that downregulation of miR-129-5p in ovarian cancer cells highly correlates with malignant progression and poor survival. Hence, we demonstrate a novel mechanism for YAP and TAZ activation in cancers, indicating not only a potentially pivotal role for miR-129-5p in the progression of ovarian cancer, but also offering new therapeutic strategies to circumvent the disease.

## INTRODUCTION

Ovarian cancer is the most lethal gynecological cancer and the fifth leading cause of cancer-related death in women [1, 2]. Despite enormous improvements in surgical techniques and therapeutic strategies, the prognosis for patients with ovarian cancer remains dismal, largely attributed to the rapid and uncontrolled tumor growth and insensitivities to chemotherapy [3, 4]. Although it is widely accepted that genetic susceptibility, environmental factors and virus infection play important roles in ovarian cancer pathogenesis, the molecular mechanism for its development and progression remains unclear [5, 6]. A better understanding of the mechanisms contributing to ovarian cell proliferation and anti-apoptosis may allow the identification of novel targets for therapeutic intervention.

The Hippo pathway represents a novel tumor-suppressor pathway and is considered to be a key regulator of tumor cell proliferation and survival [7]. YAP and TAZ are key downstream effectors of the Hippo pathway [8, 9]. When Hippo signaling is active, YAP and TAZ are phosphorylated and restricted in the cytoplasm by core complexes formed from mammalian STE20-like kinase (MST)1/2, protein salvador homolog 1 (SAV1), large tumor-suppressor kinase (LATS)1/2 and Mps one binder kinase activator-like 1 (MOB1) [9–11]. Conversely, when Hippo signaling is absent, unphosphorylated YAP1/TAZ enter the nucleus and induce the transcriptional activity of TEA domain (TEAD) family member one to four (TEAD1–TEAD4), by acting as the transcriptional co-factors [12–14]. In turn, TEAD activation promotes the transcription of genes, such as connective tissue growth factor (CTGF) and Cyclin A, leading to the promotion of cell proliferation and survival [15–17].

Constitutive activation of YAP and TAZ contributes to the development and progression of many cancers including colon, lung, liver, esophageal and ovarian cancer [18–23]. A host of factors including cell density, extracellular matrix stiffness and membrane receptors (such as G protein–coupled receptors, EGFR and leukemia inhibitory factor receptor) have been reported to influence YAP/TAZ expression and activity by modulating the Hippo pathway [24–27]. Additionally, microRNAs have demonstrated to play an important role in cancer progression by post-transcriptional suppression of multiple target mRNAs through binding to respective 3′ UTR elements [28, 29]; however, their role in regulating YAP/TAZ expression has largely remained unexplored.

Herein, we identify that the microRNA, miR-129-5p, directly represses YAP and TAZ expression, which inactivates TEAD and leads to the subsequent inhibition of ovarian cancer cell proliferation, survival and tumorigenicity. Importantly, we show that the downregulation of miR-129-5p highly correlates with ovarian cancer progression with poor prognosis. Our findings uncover a novel mechanism for YAP/TAZ overexpression, and may suggest new targets for clinical intervention in human cancers.

## RESULTS

### MiR-129-5p directly suppresses YAP and TAZ

**Figure 1:**
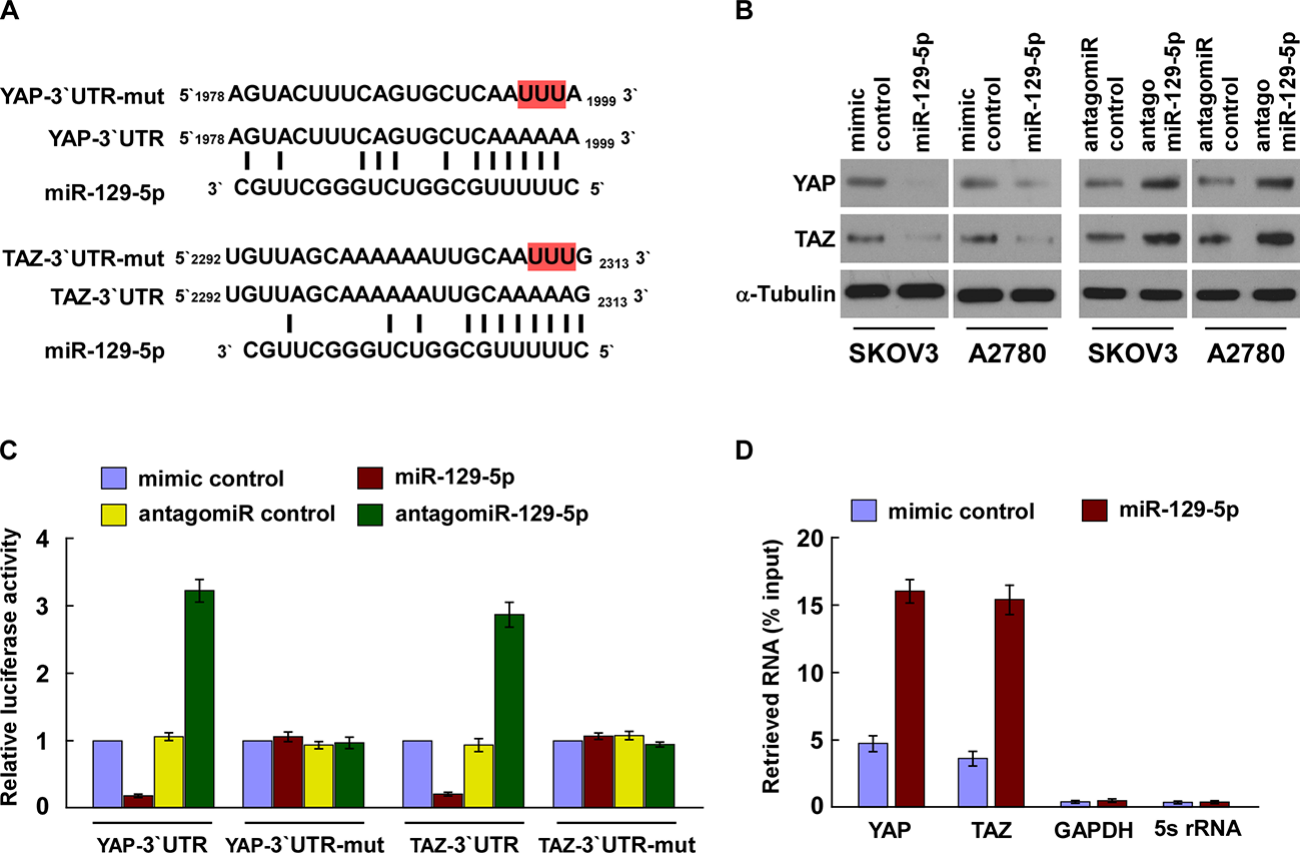
MiR-129-5p directly suppresses YAP and TAZ **(A)** Predicted miR-129-5p target sequence in 3′ UTRs of YAP and TAZ. Target sequences of YAP- and TAZ-3′ UTR were mutated. **(B)** Western blots of YAP and TAZ expression. α-Tubulin served as the loading control. **(C)** Luciferase assay of cells transfected with pGL3-YAP-3′UTR, pGL3-TAZ-3′UTR, pGL3-YAP-3′UTR-mut or pGL3-TAZ-3′UTR-mut reporter with miR-129-5p mimic, antagomiR-129-5p, mimic control or antagomiR control. **(D)** MiRNP IP assay showing the association between miR-129-5p with YAP and TAZ transcripts. *GAPDH* and 5s rRNA served as the negative controls. Each bar represents the mean ± SD of three independent experiments. *p < 0.05.

The transcriptional co-activators YAP and TAZ are major components of the Hippo signaling pathway and play a critical role in the development and progression of multiple cancer types, including ovarian cancer [21, 30]. To investigate the role microRNAs may play in regulating YAP and TAZ expression, we applied publicly available algorithms from TargetScan to reveal that YAP and TAZ might be potential targets of miR-129-5p (Figure 1A). Our western blot analysis revealed that expression levels of YAP and TAZ were significantly decreased in miR-129-5p-transfected cells, but increased in miR-129-5p-silenced cells (Figure 1B and Supplementary Figure 1A–1B), suggesting that miR-129-5p negatively regulates these two proteins. However, significant changes to YAP and TAZ expression were not observed when cells were transfected with miR-101, miR-124 and miR-18a, which are also predicted to target YAP and TAZ, and miR-129-5p has no effect on the LATS1 phosphorylation level (Supplementary Figure 1C), suggesting that YAP and TAZ are selectively regulated by miR-129-5p (data not shown). Furthermore, luciferase assays revealed miR-129-5p overexpression led to a decrease in reporter activity linked with the 3′ UTRs of YAP and TAZ transcripts, while in miR-129-5p silenced cells, an increase in reporter activity was observed (Figure 1C). However, neither overexpressing miR-129-5p nor silencing miR-129-5p exhibited effects on the reporter activities linked with mutant 3′ UTRs of YAP and TAZ. Importantly, micro-ribonucleoprotein (miRNP) immunoprecipitation (IP) assay showed that miR-129-5p overexpression enriched the transcripts of YAP and TAZ (and not GAPDH or 5s rRNA, that assembled into the miRNP complexes, indicating that miR-129-5p directly targets the mRNA 3′ UTR regions of these transcripts (Figure 1D). Taken together, our results demonstrate that YAP and TAZ are *bona fide* targets of miR-129-5p.

### MiR-129-5p inactivates TEAD activity

**Figure 2:**
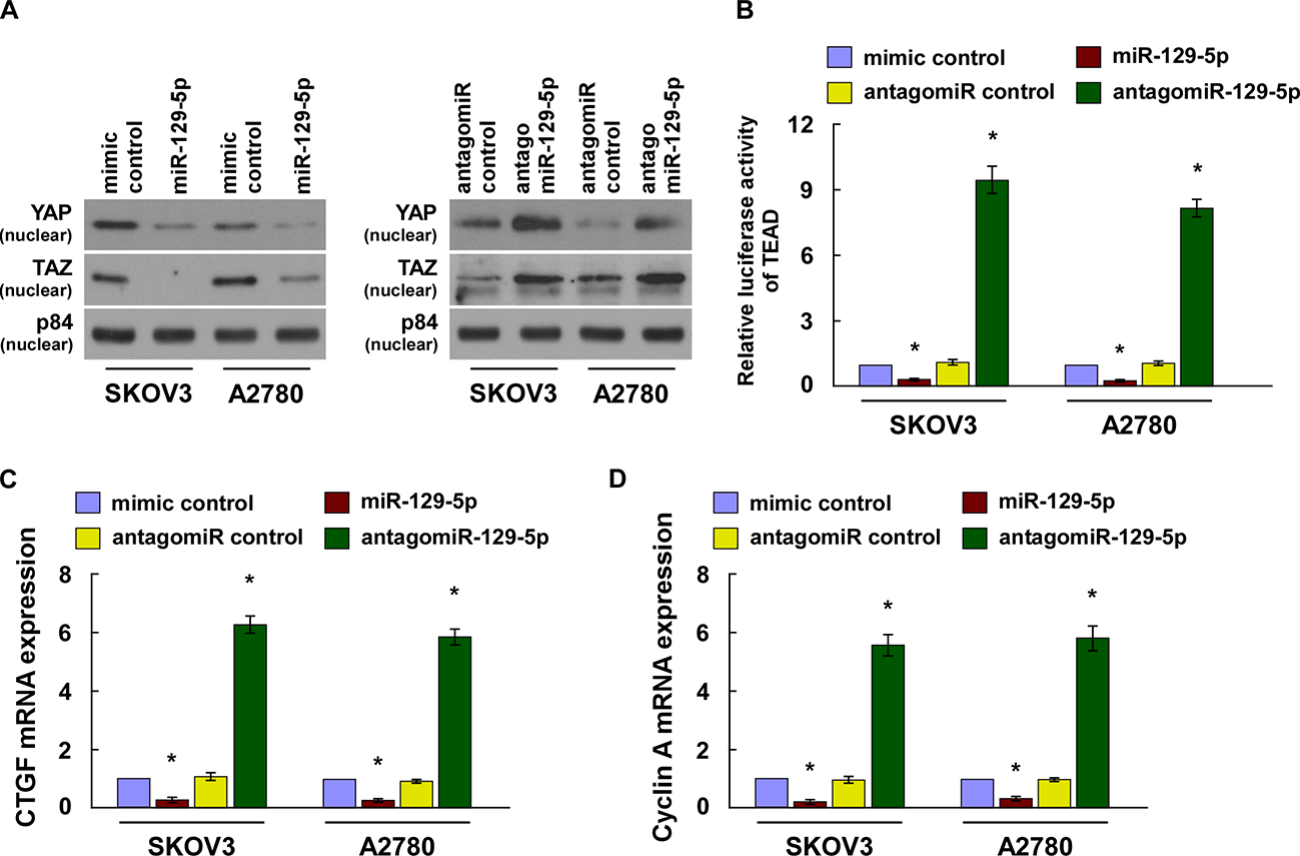
MiR-129-5p inactivates the Hippo signaling pathway **(A)** Western blotting of nuclear YAP and TAZ expression. The nuclear protein p84 was used as a nuclear protein marker. **(B)** TEAD transcriptional activity was assessed in the indicated cell lines by TEAD dependent promoter-driven firefly luciferase reporter construct. **(C and D)** Real-time PCR analysis of CTGF and Cyclin A in indicated cells. Transcript levels were normalized to *GAPDH* expression. Each bar represents the mean ± SD of three independent experiments. *p < 0.05.

YAP or TAZ activates TEAD transcription through nuclear translocation and interaction with TEAD transcription factors [12, 14]. Our cellular fractionation and western blot assays revealed that when overexpressing miR-129-5p reduced nuclear accumulation of YAP and TAZ, while when silencing miR-129-5p upregulated nuclear YAP and TAZ expression (Figure 2A). Moreover, overexpression of miR-129-5p significantly reduced TEAD dependent luciferase activity, levels of CTGF and Cyclin A, and binding capability of TEAD with *CTGF* and *Cyclin A* promoter in ovarian cancer cells. MiR-129-5p silencing increased such effects (Figure 2B–2D and Supplementary Figure 2).

### MiR-129-5p inhibits ovarian cancer cell proliferation and survival *in vitro*

**Figure 3:**
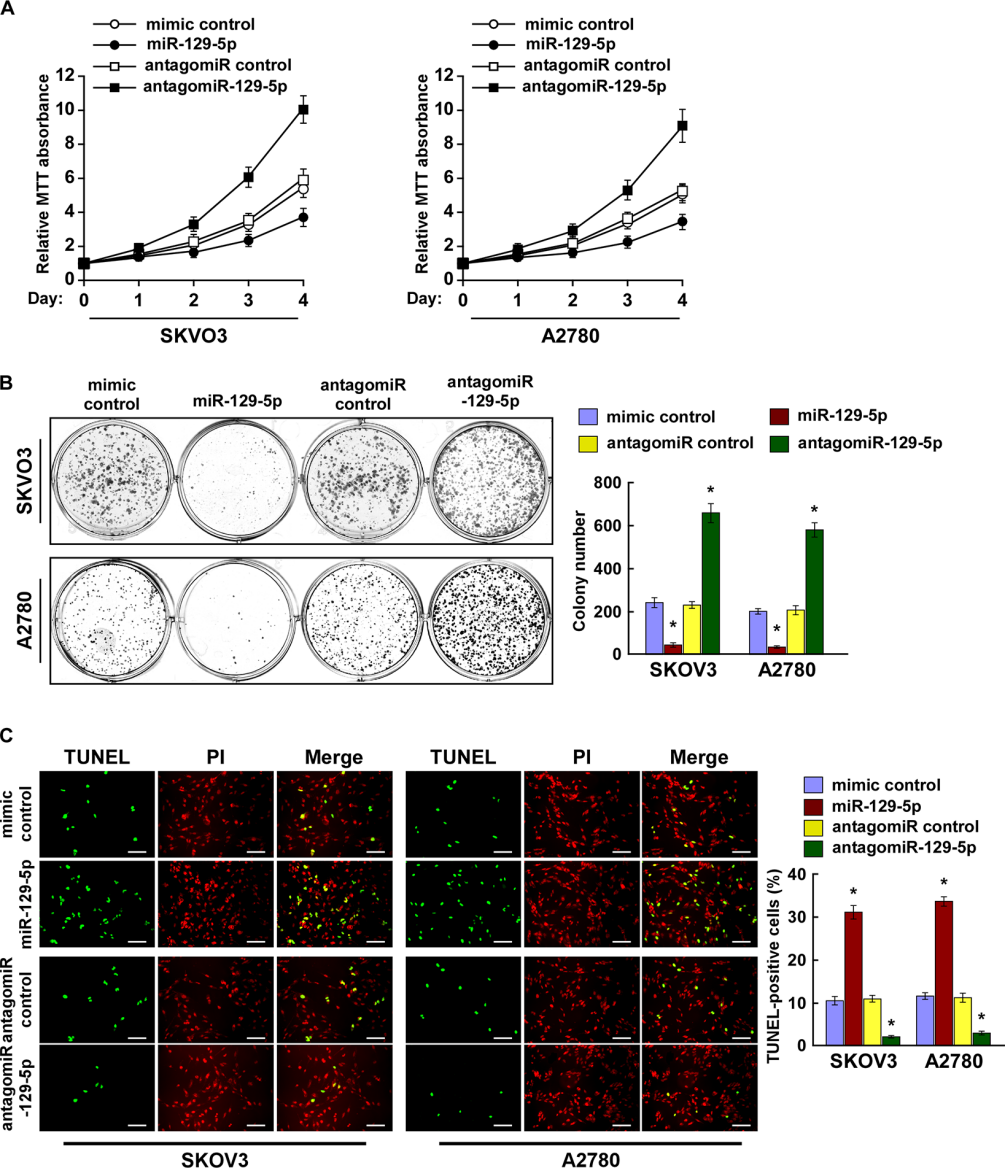
MiR-129-5p inhibits ovarian cancer cell proliferation and survival *in vitro* **(A)** MTT assay reveals that miR-129-5p upregulation inhibits, while miR-129-5p inhibition promotes, proliferation of SKOV3 and A2780 cells. **(B)** Representative micrographs (left) and quantification (right) of crystal violet–stained cell colonies. **(C)** Representative micrographs (left) and quantification of TUNEL-positive cells in cells treated with cisplatin (20 μM) for 36 h. Scale bars: 50 μm. Each bar represents the mean of three independent experiments. *p < 0.05.

Hippo pathway inactivation and the subsequent nuclear translocations of YAP and TAZ have been shown to promote cell proliferation and resistance to death [31, 32]. As expected, MTT and colony formation assays showed that miR-129-5p upregulation significantly decreased the proliferation rate of the SKOV3 and A2780 cells, while miR-129-5p inhibition increased proliferation rates (Figure 3A and 3B). In addition, our TUNEL assays demonstrated that ectopic expression of miR-129-5p rendered ovarian cancer cells more sensitive to treatment by the chemotherapeutic agent cisplatin (Figure 3C). Therefore, our results suggest miR-129-5p inhibits ovarian cancer cell proliferation and survival *in vitro*.

### YAP and TAZ are functionally involved in miR-129-5p-mediated ovarian cancer cell proliferation and survival

**Figure 4:**
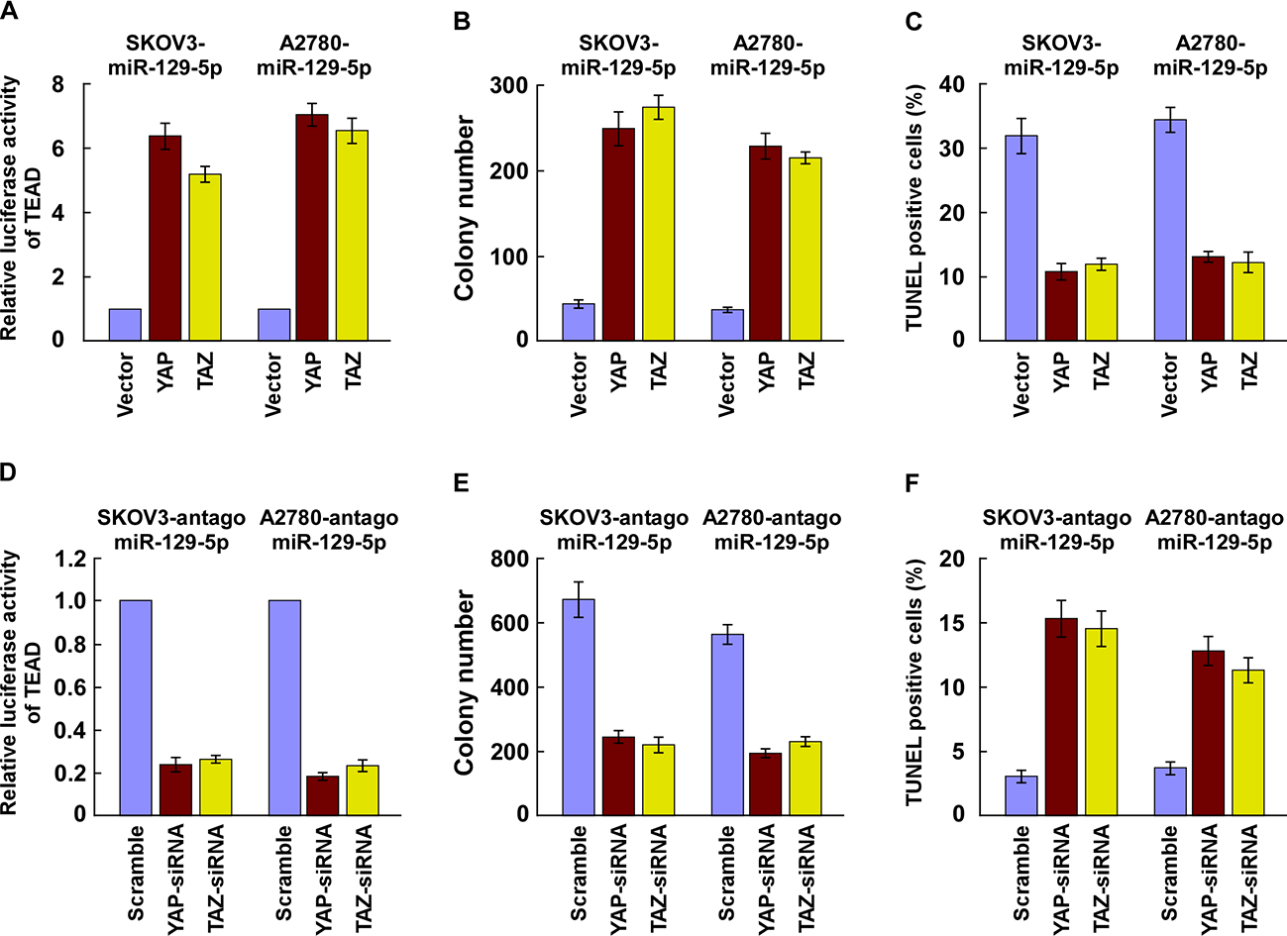
YAP and TAZ are functionally involved in miR-129-5p-mediated ovarian cancer cell proliferation and survival **(A)** miR-129-5p-induced suppression of TEAD transcriptional activity is reversed by expression of either YAP or TAZ in indicated cells. **(B and C)** Suppressive effects of miR-129-5p on ovarian cell proliferation (B) and survival (C) are antagonized by expression of YAP or TAZ. **(D)** TEAD transcriptional activity induced by antagomiR-129-5p is suppressed by depletion of either YAP or TAZ in indicated cells. **(E and F)** Promotion of ovarian cell proliferation (E) and survival (F) induced by antagomiR-129-5p is inhibited by YAP or TAZ silencing.

We further explored the functional significance of YAP and TAZ in cell proliferation and survival of ovarian cancer cell lines, in addition to TEAD activity induced by miR-129-5p. As shown in Figure 4A and Supplementary Figure 3A–3C, reintroduction of YAP/TAZ abrogated the suppression of TEAD transcriptional activity, CTGF and Cyclin A expression. Moreover, when YAP/TAZ or their 3′UTR elements was ectopically overexpressed in miR-129-5p-transduced SKOV3 and A2780 cells, miR-129-5p-induced cell growth arrest and death were, in part, antagonized (Figure 4B and 4C and Supplementary Figure 3B). Depletion of YAP or TAZ dramatically suppressed TEAD transcriptional activity in miR-129-5p silenced ovarian cells (Figure 4D and Supplementary Figure 3D). Furthermore, silencing of YAP or TAZ suppressed the proliferation rate and survival of antagomiR-129-5p-transfected SKOV3 and A2780 cells (Figure 4E and 4F). These results suggest that YAP and TAZ are functionally relevant effectors of miR-129-5p in ovarian cancer cell proliferation and survival.

### miR-129-5p suppresses tumorigenicity of ovarian cancer cells *in vivo*

**Figure 5:**
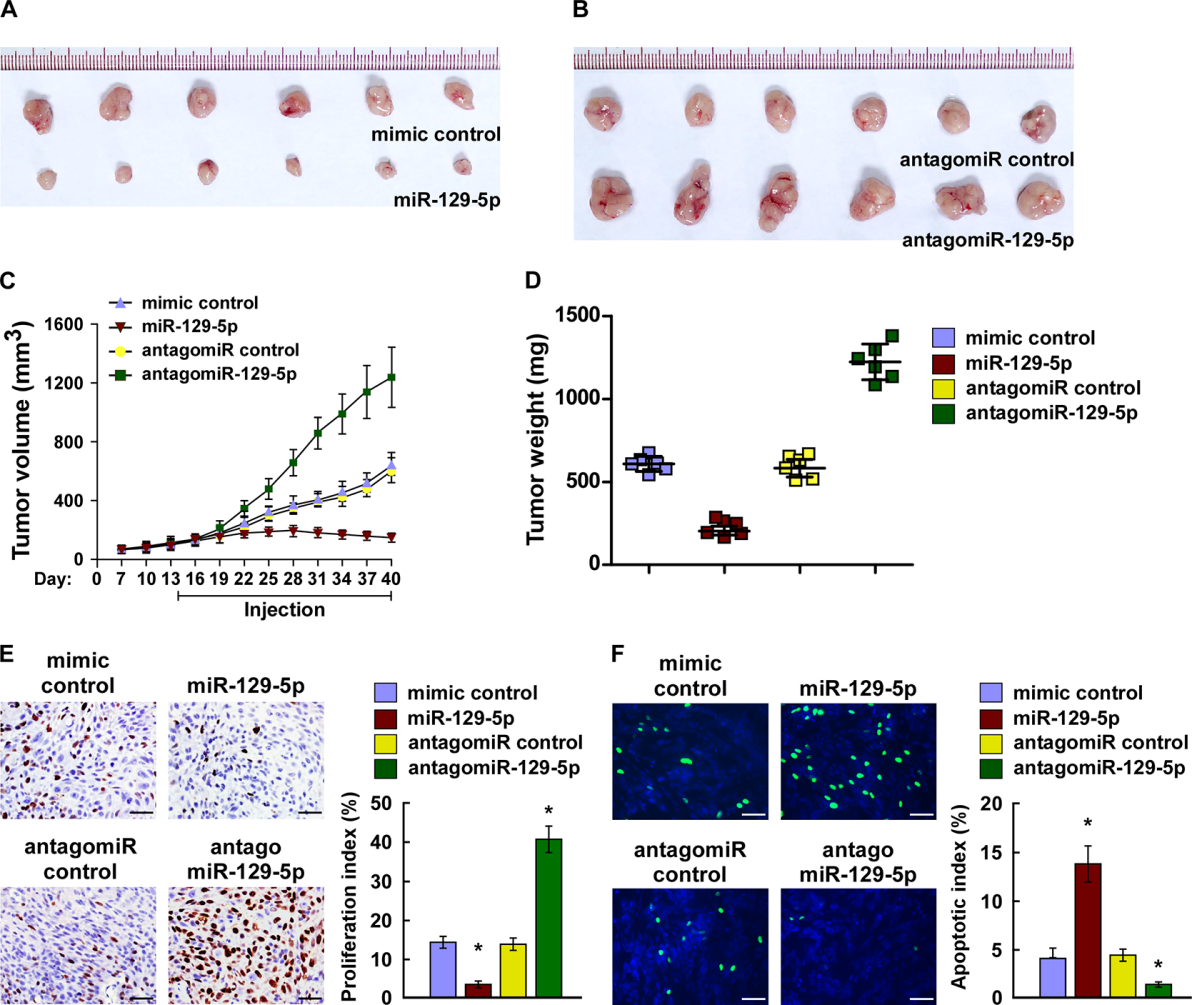
miR-129-5p suppresses tumorigenicity of ovarian cancer cells *in vivo* **(A and B)** Xenograft model in nude mice. Images of tumors from all mice in each group. **(C)** Tumor volumes were measured on the indicated days and presented as the mean ± SD. **(D)** Tumor weights of each group. **(E)** Proliferation index determined by counting the proportion of Ki67-positive cells. **(F)** Apoptotic index measured by the percentage of TUNEL-positive cells. Scale bars: 50 μm. Each bar represents the mean ± SD of three independent experiments. *p < 0.05.

We then examined the tumor suppressive role of miR-129-5p in ovarian cancer progression using an *in vivo* tumor model. Importantly, intratumoral injection with miR-129-5p mimic dramatically inhibited tumor growth, while injecting a mimic control had no effect on tumor development (Figure 5A, and 5D). Conversely, the tumors injected with antagomiR-129-5p were significantly larger, in both size and weight, than the control tumors (Figure 5B–5D). The expression of miR-129-5p is marked upregulated in the miR-125-5p/tumors but decreased in the antagomiR-125-5p/tumors compared control tumors, respectively (Supplementary Figure 4A). Furthermore, western blotting analysis showed that overexpressing miR-129-5p reduced, while silencing miR-129-5p increased YAP and TAZ in xenograft tumors, further supporting the notion that miR-129-5p regulated the tumor growth via YAP and TAZ (Supplementary Figure 4B). Meanwhile, our staining assays revealed that miR-129-5p-overexpressing tumors exhibited decreased Ki67-positive cells and increased TUNEL-positive cells, whereas miR-129-5p-silenced tumors presented a higher Ki67 proliferation index and decreased TUNEL-positive apoptotic cells (Figure 5E and 5F). Here, our results suggest that miR-129-5p suppresses the tumorigenicity of ovarian cancer cells *in vivo*.

### Downregulation of miR-129-5p correlates with ovarian cancer progression

**Figure 6:**
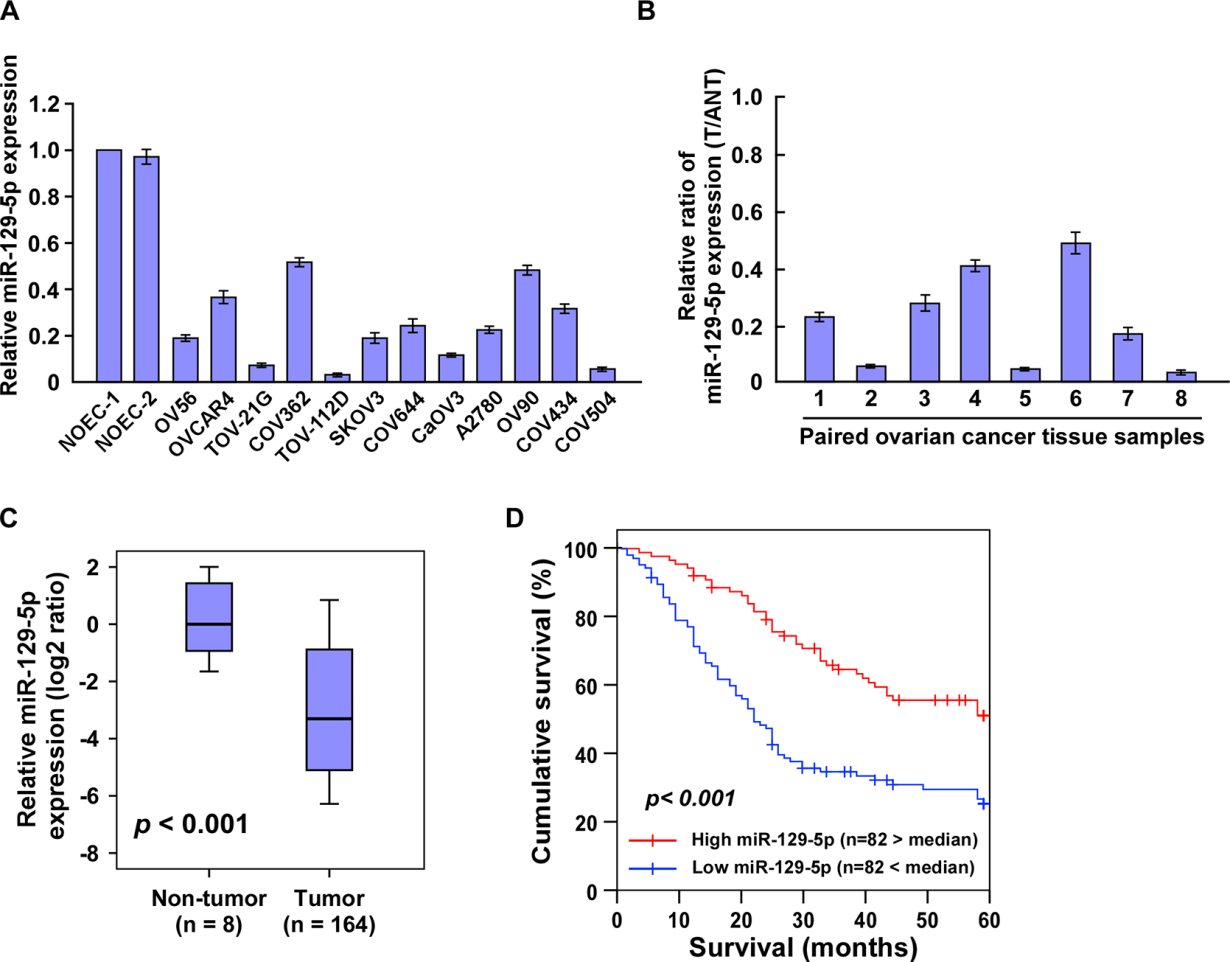
Downregulation of miR-129-5p correlates with ovarian cancer progression **(A and B)** Real-time PCR analysis of miR-129-5p expression in (A) two NOEC (normal ovarian epithelial cell) lines and 12 cultured ovarian cancer cell lines, and (B) eight pairs of ovarian cancer samples (T) and adjacent normal ovary tissues (ANT). Transcript levels were normalized to *U6* expression. **(C)** MiR-129-5p expression in 164 ovarian cancer samples and eight normal ovarian tissues assessed by real-time PCR. Transcript levels were normalized to *U6* expression. Boundaries of boxes represent lower and upper quartiles, respectively. Lines within boxes and whiskers denote median and extremum, respectively. **(D)** Kaplan–Meier analysis of 5-year overall survival curves of ovarian cancer patients with low miR-129-5p expression (< median, *n* = 82) or high miR-129-5p expression (> median, *n* = 82). Each bar represents the mean ± SD of three independent experiments. *p < 0.05.

We next evaluated whether the expression of miR-129-5p is clinically correlated with ovarian cancer progression. Real-time PCR analysis revealed that miR-129-5p was differentially downregulated in 12 tested ovarian cancer cell lines when compared with two normal ovarian epithelial cell (NOEC) lines; and in eight primary ovarian cancer tissues when compared with the paired tumor-adjacent tissues (Figure 6A and 6B), implying that miR-129-5p is downregulated in human ovarian cancer.

We then examined miR-129-5p expression in archived ovarian cancer specimens. Figure 6C shows that miR-129-5p was markedly decreased in ovarian cancer samples when compared with 10 normal ovarian samples. In the ovarian cancer samples, statistical analysis revealed that decreased miR-129-5p expression strongly correlated with FIGO stage (*p* = 0.004), histological differentiation (*p* < 0.001) and pelvic metastasis (*p* = 0.006) (Supplementary Table 1 and 2). Importantly, we identify miR-129-5p expression as an independent prognostic factor in patients with ovarian cancer (hazard ratio = 2.365, 95% CI = 1.831–2.952, *p* < 0.001; Supplementary Table 3), and show low miR-129-5p expression is associated with shorter overall survival in patients with primary ovarian cancer (*p* < 0.001; Figure 6D). Taken together, these results suggest that miR-129-5p downregulation is involved in human ovarian cancer progression.

### Clinical relevance of miR-129-5p, YAP and TAZ in ovarian cancer

**Figure 7:**
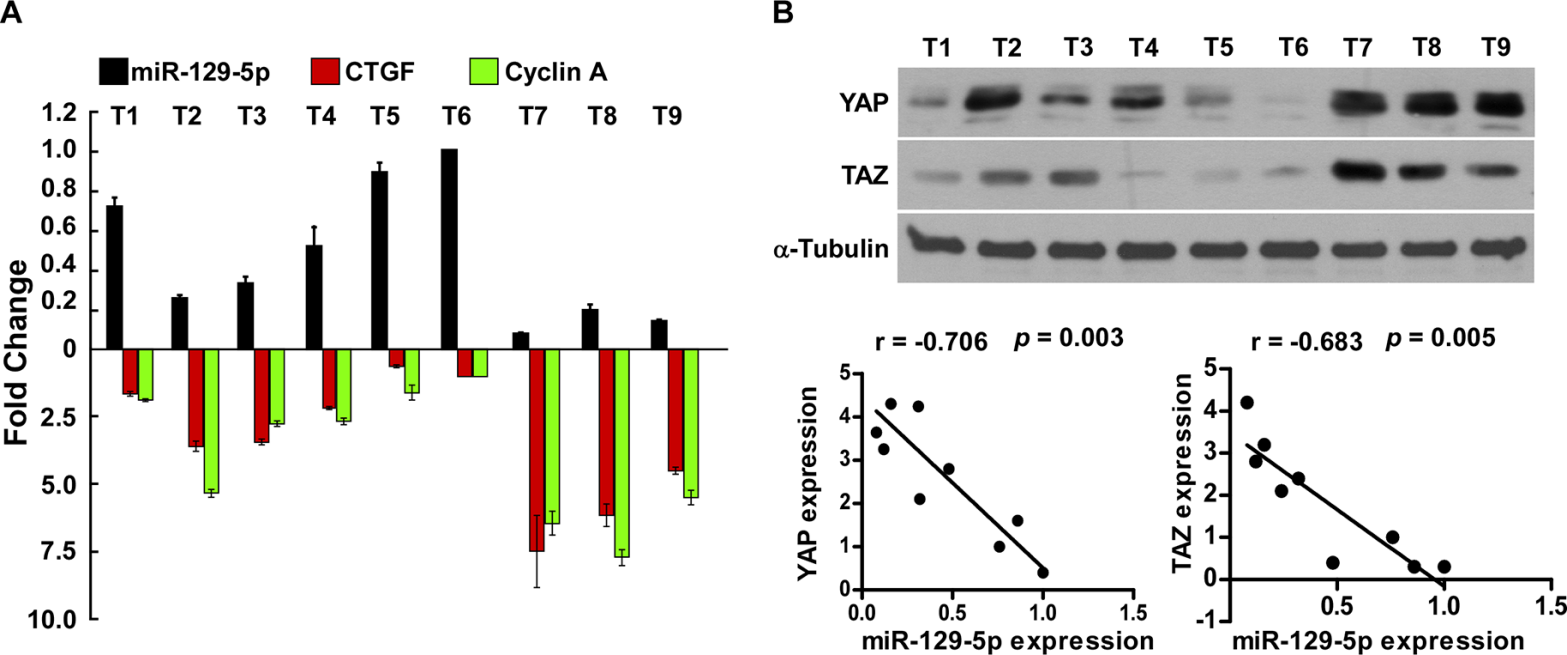
Clinical relevance of miR-129-5p, YAP, TAZ, CTGF and Cyclin A in ovarian cancer **(A and B)** Expression analysis (A) and correlation (B) of miR-129-5p expression and YAP, TAZ protein expression, and *CTGF*, *Cyclin A* mRNA levels in nine freshly collected human ovarian cancer tissue samples (T) The ratio of first sample (YAP/α-tubulin, TAZ/α-tubulin) was considered as 1.0. α-Tubulin were used as loading controls. Each bar represents the mean ± SD of three independent experiments.

Finally, we examined whether miR-129-5p-mediated suppression of YAP and TAZ in ovarian cancers are clinically relevant. As shown in Figure 7A–7B and Supplementary Figure 5, miR-129-5p levels in nine freshly collected ovarian cancer samples were inversely correlated with the expression levels of YAP (*r* = –0.706, *p* = 0.003) and TAZ (*r* = –0.683, *p* = 0.005), and mRNA levels of Hippo downstream genes CTGF (*r* = –0.832, *p* < 0.001) and Cyclin A (*r* = –0.801, *p* < 0.001). Our results suggest that miR-129-5p downregulation increases YAP and TAZ expression, consequently resulting in an aggressive ovarian cancer with poor prognosis.

## DISCUSSION

YAP and TAZ transcriptional co-factors are key downstream effectors of the Hippo signaling pathway and play a crucial role in various cellular processes [8, 9]. Physiologically, the activities of YAP and TAZ are largely restricted by the core complex of the Hippo pathway, and terminated by sequential phosphorylation, inhibition of nuclear translocalization and degradation [9–11]. Activation of YAP and TAZ have been observed in many human diseases, including cancers [18–23]. A series of factors including cell density, extracellular matrix stiffness, G protein–coupled receptors, EGFR and leukemia inhibitory factor receptors have been reported to regulate YAP/TAZ expression and activity by modulating the Hippo pathway [24–27]. Moreover, microRNAs have been recently shown to regulate the Hippo signaling pathway by acting upstream or downstream of YAP and TAZ [33–36]. However, whether YAP and TAZ are directly regulated by microRNAs in human cancers have until now remained unclear. In this study, we demonstrate that miR-129-5p directly inhibits YAP and TAZ expression, which leads to the inactivation of TEAD and subsequent inhibition of ovarian cancer cell proliferation, survival and tumorigenicity. Consistent to our observations, we find miR-129-5p is substantially downregulated in ovarian cancer cells. Altogether, our findings reveal a novel mechanism for YAP and TAZ activation in ovarian cancer.

Through bioinformatics analysis, we reveal oncogenes YAP and TAZ are indicated as theoretical miR-129-5p targets. In turn, several lines of evidence demonstrate that YAP and TAZ are *bona fide* targets of miR-129-5p. First, western blot analysis showed that miR-129-5p decreased expression levels of YAP and TAZ. Second, the luciferase activity assay with 3′ UTR and the miRNP IP assay revealed miR-129-5p repressed YAP and TAZ expression via interaction with YAP- and TAZ-3′ UTR elements. Third, overexpression of miR-129-5p inhibited TEAD transcriptional activity and expression of CTGF and Cyclin A, while downregulation of miR-129-5p promoted such activities. Finally, expression of YAP and TAZ antagonized the effect of miR-129-5p on cell proliferation and survival.

Owing to its pleiotropic functions in cancer development and progression, YAP and TAZ have emerged as important targets for cancer therapy [37–39]. Unlike most current oncogenic signaling cascades, the majority of kinases upstream of YAP and TAZ are tumor suppressors, suggesting that the conventional approach of designing small-molecule kinase inhibitors is unlikely to work [37–39]. Our findings introduce the inhibition of YAP and TAZ as a novel strategy in cancer therapy.

Of note, microRNAs have been characterized as important regulatory mechanisms of gene expression. Multiple microRNAs have already progressed into clinical development, such as the miR-34a mimic compound, MRX34, which has successfully advanced into a Phase I human clinical trial for liver cancer [40]. In this study, we demonstrated that miR-129-5p robustly downregulates endogenous YAP and TAZ expression and inhibits ovarian cancer cell proliferation and survival. Importantly, the delivery of miR-129-5p mimic dramatically inhibited the tumorigenicity of ovarian cancer cells, suggesting the anti-cancer potential of the miR-129-5p mimic.

Interestingly, by analysis of the promoter region of miR-129-5p using the CONSITE program, we found that miR-129-5p was potentially targeted by p53, whose malfunction is frequently detected in human cancers. Meanwhile, multi-CpG-rich loci of TRPM3 promoter were retrieved by the UCSC genome browser, suggesting that miR-129-5p downregulation might be associated with genomic methylation. Thus, it would be of great interest to further investigate whether downregulation of miR-129-5p in ovarian cancer is attributed to genomic methylation and/or p53-mediated transcriptional regualtion.

In summary, our study has revealed that miR-129-5p downregulation plays an important role in ovarian cancer progression and that miR-129-5p is a critical repressor of YAP and TAZ. Understanding the precise role of miR-129-5p in the pathogenesis of ovarian cancer and activation of the YAP/TAZ signaling pathway promises to increase our knowledge of the biological basis of cancer development, and may also allow the development of new therapeutic strategies against ovarian cancer.

## MATERIALS AND METHODS

### Cells, tissue specimens and clinicopathological characteristics

The ovarian cancer cell lines OV56, OVCAR4, TOV-21G, COV362, TOV-112D, SKOV3, COV644, CaOV3, A2780, OV90, COV434 and COV504 were maintained in RPMI 1640 (Invitrogen, Carlsbad, CA, USA) supplemented with 10% FBS (HyClone, Logan, UT, USA).

The 164 paraffin-embedded, archived ovarian cancer samples used in this study were histopathologically and clinically diagnosed at the the First Affiliated Hospital of Sun Yat-sen University between 2008 and 2011. The disease stages of all the patients were classified according to the International Federation of Gynecology and Obstetrics (FIGO) guidelines for clinical staging. Written informed consent was obtained from all patients prior to the study. The Institutional Research Ethics Committee approved the use of the clinical specimens for research purposes. The clinicopathological characteristics of the samples are summarized in Supplementary Table 1. Freshly collected ovarian cancer tissue specimens and matched adjacent non-tumour ovarian tissue specimens were each collected from 8 patients, and were frozen and stored in liquid nitrogen until used. The correlation between miR-129-5p and the target genes was determined using another 9 freshly collected ovarian cancer tissues.

### Plasmid, oligonucleotides, and transfection

The human miR-129-5p gene was PCR-amplified from genomic DNA and cloned into a pMSCV-puro retroviral vector. The TEAD Luciferase Reporting system was purchased from lifeome (Oceanside, CA, USA). We purchased miR-129-5p mimic, miR-129-5p antagonist (antagomiR-129-5p), and controls from RiboBio (Guangzhou, China). Transfection of the plasmids, siRNAs, miR-129-5p mimic, and antagomiR-129-5p were performed using Lipofectamine 2000 (Invitrogen) according to the manufacturer's instructions.

### Western blotting

Cells were harvested in cell lysis buffer (Cell Signaling Technology, Danvers, MA, USA) and heated for 5 min at 100°C. Equal quantities of denatured protein samples were resolved on 10% SDS-polyacrylamide gels, and then transferred onto PVDF membranes (Roche, Basel, Switzerland). After blocking with 5% non-fat dry milk in TBS/0.05% Tween 20, membranes were incubated with a specific primary antibody, followed by a horseradish peroxidase-conjugated secondary antibody. Proteins were visualised using ECL reagents (Pierce, Rockford, IL, USA). Antibodies against YAP, TAZ and p84 were purchased from Abcam (Cambridge, MA, USA). The membranes were stripped and reprobed with an anti-α-tubulin antibody (Sigma-Aldrich, St. Louis, MO, USA) as the loading control.

### MiRNA extraction and real-time quantitative PCR

Total miRNA from cultured cells and fresh surgical ovarian tissues was extracted using a mirVana miRNA Isolation Kit (Ambion, Austin, TX, USA) according to the manufacturer's instructions. We synthesised cDNA from 10 ng total RNA using a TaqMan miRNA reverse transcription kit (Applied Biosystems, Foster City, CA, USA), and quantified the expression levels of miR-129-5p using a miRNA-specific TaqMan MiRNA Assay Kit (Applied Biosystems). The expression of miRNA was defined based on the Ct, and relative expression levels were calculated as 2^−[(Ct of miR-129-5p)–(Ct of U6)]^ after normalisation with reference to expression of U6 small nuclear RNA.

### miRNP immunoprecipitation

miR-129-5p-overexpressing and control cells were transfected with HA-Ago1, followed by HA-Ago1 immunoprecipitation using HA-antibody. Real-time PCR analysis of the IP material was used to test the association of the mRNA of YAP and TAZ with the RISC complex. The GAPDH gene and 5s rRNA were used as a negative controls.

### Tunel

Apoptotic DNA fragmentation was examined using an *in situ* DeadEnd™ Fluorometric TUNEL System assay kit (Promega, Madison, WI, USA) according to the manufacturer's protocol. Briefly, cells were plated in 24-well flat-bottom plates and treated with cisplatin (10 μM) for 36 h. Cells were fixed in 4% paraformaldehyde at 4°C for 30 min, permeabilised in 0.1% Triton X-100, and labelled with fluorescein-12-dUTP using terminal deoxynucleotidyl transferase. The localised green fluorescence of apoptotic cells from the fluorescein-12-dUTP was detected by fluorescence microscopy (Axiovert 100M, Zeiss, Oberkochen, Germany).

### Xenografted tumor model and staining

BALB/c-nu mice (5–6 weeks old, 18–20 g) were purchased from the Experimental Animal Center of the Guangzhou University of Chinese Medicine and housed in barrier facilities on a 12-h light/dark cycle. The Institutional Animal Care and Use Committee of Sun Yat-sen University approved all experimental procedures. The mice were randomly assigned to groups (*n* = 6/group). The mice in groups were inoculated subcutaneously with SKOV3 cells (5 × 10^6^) in the left dorsal flank, and two weeks later, injected intratumorally with 100 μL miR-129-5p mimic, mimic control, antagomiR-129-5p control or antagomiR control (diluted in PBS at 2 mg/mL) three times per week for three weeks. Tumors were examined twice weekly; length, width, and thickness were measured with callipers, and tumor volumes were calculated. Tumor volume was calculated using the equation (L × W^2^)/2. On day 40, the animals were euthanised, and the tumors were excised, weighed, and paraffin-embedded. Serial 6.0-μm sections were cut and subjected to staining assays. The proliferation index was determined by counting the proportion of Ki67-positive cells. The apoptotic index was measured based on the percentage of TUNEL-positive cells.

### Luciferase assays

Cells (4 × 10^4^) were seeded in triplicate in 24-well plates and cultured for 24 h. Cells were transfected with 100 ng TEAD reporter luciferase plasmid [12], or pGL3-YAP-3′UTR, or pGL3-TAZ-3′UTR luciferase plasmid, plus 5 ng pRL-TK Renilla plasmid (Promega) using Lipofectamine 2000 (Invitrogen) according to the manufacturer's recommendation. Luciferase and Renilla signals were measured 36 h after transfection using a Dual Luciferase Reporter Assay Kit (Promega) according to the manufacturer's protocol.

### Nuclear/cytoplasmic fractionation

Cells were washed with cold PBS and resuspended in buffer containing 10 mM HEPES (pH 7.8), 10 mM KCl, 0.1 mM EDTA, 1 mM Na_3_VO_4_, 1 mM DTT, 1:500 protease inhibitors (Sigma-Aldrich), and 0.2 mM PMSF, and incubated on ice for 15 min. Detergent was added and cells were vortexed for 10 s at the highest setting. Nuclei and the supernatant (“cytoplasm”) were separated by centrifugation at 4°C. Nuclei were resuspended in buffer containing 20 mM HEPES (pH 7.8), 0.4 M NaCl, 1 mM EDTA, 1 mM Na_3_VO_4_, 1 mM DTT, and 1:500 protease inhibitors and incubated on ice for 15 min. Nuclear extracts were collected by centrifugation at 14,000 × *g* for 10 min at 4°C.

### Statistical analysis

All statistical analyses were carried out using SPSS 13.0 statistical software (SPSS Inc., Chicago, IL, USA). Survival curves were plotted using the Kaplan–Meier method and compared by log-rank test. The 2-tailed Student's *t*-test was used to evaluate the significance of differences between two groups of data in all pertinent experiments. A *p*-value < 0.05 was considered significant.

## SUPPLEMENTARY MATERIALS AND METHODS


