# Supplementary material for: A novel role for microRNA-129-5p in inhibiting ovarian cancer cell proliferation and survival via direct suppression of transcriptional co-activators YAP and TAZ
Source: Oncotarget. 2015 Apr 7;6(11):8676–86. doi: 10.18632/oncotarget.3254 (PMC4496175; doi:10.18632/oncotarget.3254)
Supplement: Supplementary file 1 [file oncotarget-06-8676-s001.pdf]

SUPPLEMENTARY MATERIALS AND METHODS

Primers and siRNAs

CTGF-up: 5′-CAGGCTAGAGAAGCAGAGCC-3′; CTGF-dn: 5′-TGGAGATTTTGGGAG TACGG-3′; Cyclin A-up: 5′-CAGAAAACCATTGGTCCCTC-3′ Cyclin A-dn: 5′-CACTC ACTGGCTTTTCATCTTC-3′; GAPDH-up: 5′-ATTCCACCCATGGCAAATTC-3′; GAPDH-dn: 5′-TGGGATTTCCATTGATGACAAG-3′.

miRNP primers: GAPDH-up: 5′-TCCCCCACCACACTGAATCT-3′; GAPDH-dn: 5′-GCCCCTCCCCTCTTCAAG-3′; 5S rRNA-up: 5′-ATCTCGTCTGATCTCGGAAGCT-3′; 5S rRNA-dn: 5′-AGGCGGTCTCCCATCCA-3′; YAP-up: 5′-TTGCCAGTAGCCACAGA TTAA-3′; YAP-dn: 5′-CCCAAACTTTCCCACCCTC-3′; TAZ-up: 5′-TTGAGGGTGTATG GTGGAGA-3′; TAZ-dn: 5′-AACTGTAGCAAACAGGATTAGGA-3′;

YAP siRNA: 5′-CAGGUGAUACUAUCAACCAAA-3′; TAZ siRNA: 5′-CCUGCCGGAGU CUUUCUUUAA-3′;

SUPPLEMENTARY FIGURES AND TABLES

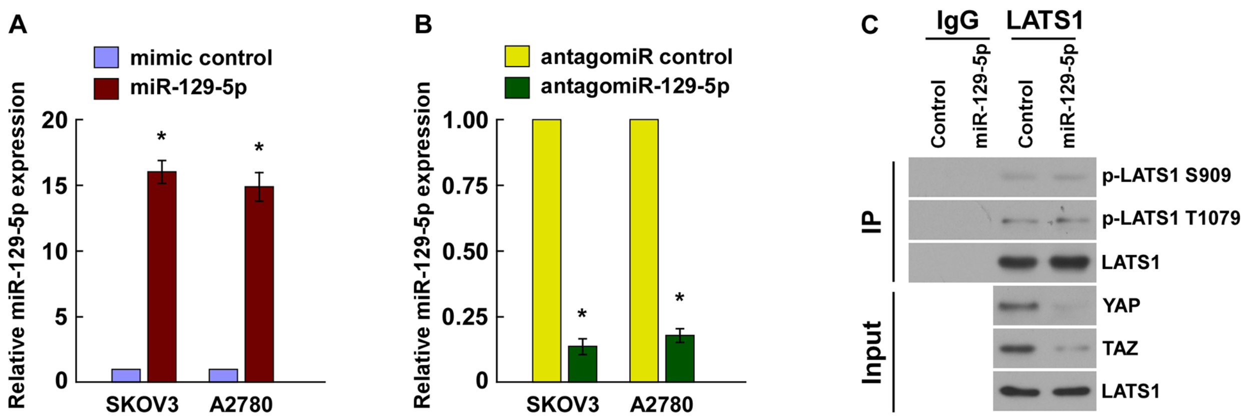

Supplementary Figure 1: (A and B) Real-time PCR analysis of miR-129-5p expression in miR-129-5p-overexpressing and -silenced cells. Transcript levels were normalised by *U6* expression. Each bar represents the mean of three independent experiments. *$p < 0.05$. **(C)** Cell lysates from control or miR-129-5p-overexpressing cells were divided into two parts, one for IgG IP and the other for LATS1 IP. Endogenous LATS1 was immunoprecipitated and probed with phospho-specific antibodies.

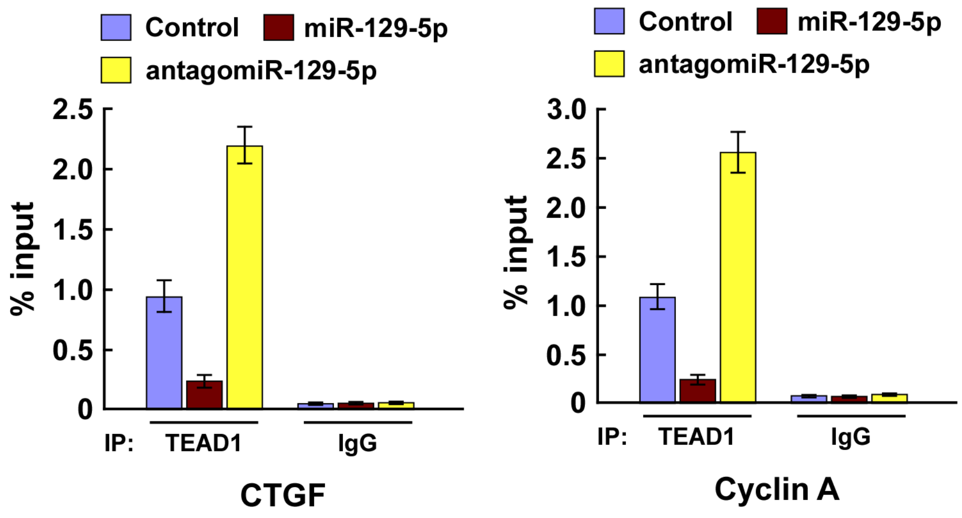

Supplementary Figure 2: A ChIP assay revealed that overexpression of miR-129-5p reduced, while silencing miR-129-5p increased, the binding capability of TEAD with *CTGF* and *Cyclin A* promoter.

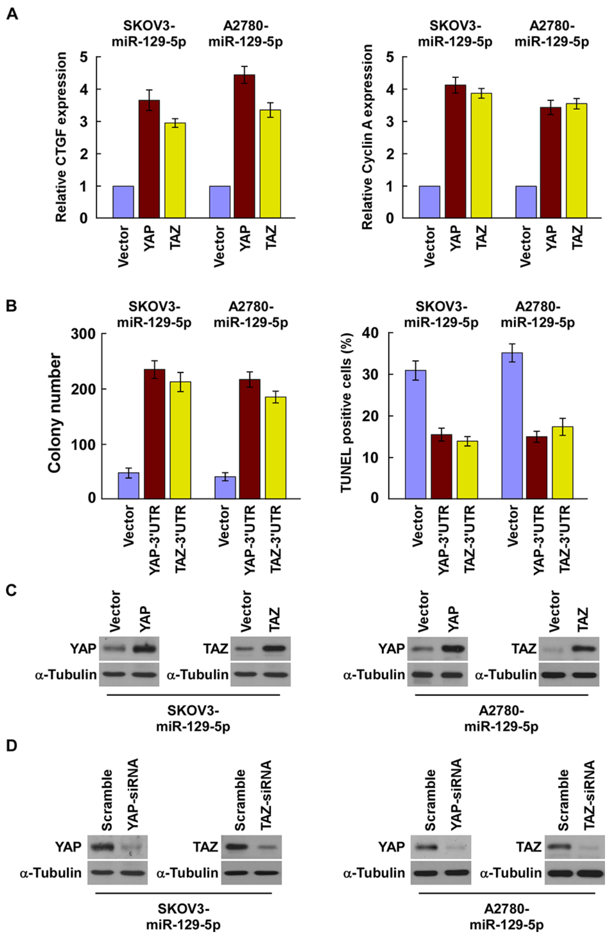

Supplementary Figure 3: (A) Real-time PCR analysis of CTGF and Cyclin A in indicated cells. Transcript levels were normalized to *GAPDH* expression. (B) Overexpression of YAP-3′UTR or TAZ-3′UTR elements antagonized miR-129-5p effects on SKOV3 and A2780 cell proliferation and survival. Each bar represents the mean ± SD of three independent experiments. *$p < 0.05$. **(C and D)** Western blot analysis of YAP and TAZ in indicated cells.

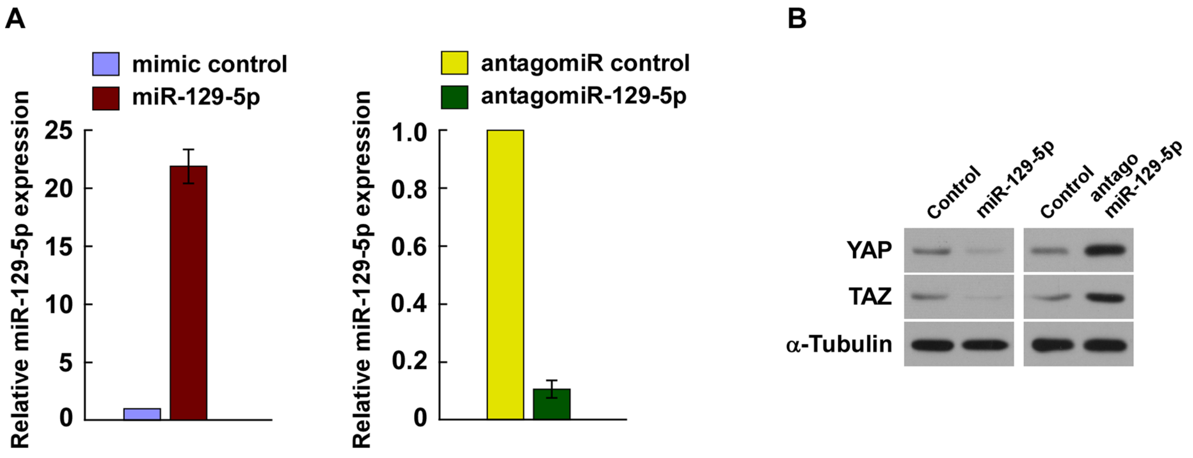

Supplementary Figure 4: (A) The expression of miR-129-5p is marked upregulated in the miR-125-5p/tumors but decreased in the antagomiR-125-5p/tumors compared control tumors, respectively. (B) Western blotting analysis showed that overexpressing miR-129-5p reduced, while silencing miR-129-5p increased YAP and TAZ in xenograft tumors.

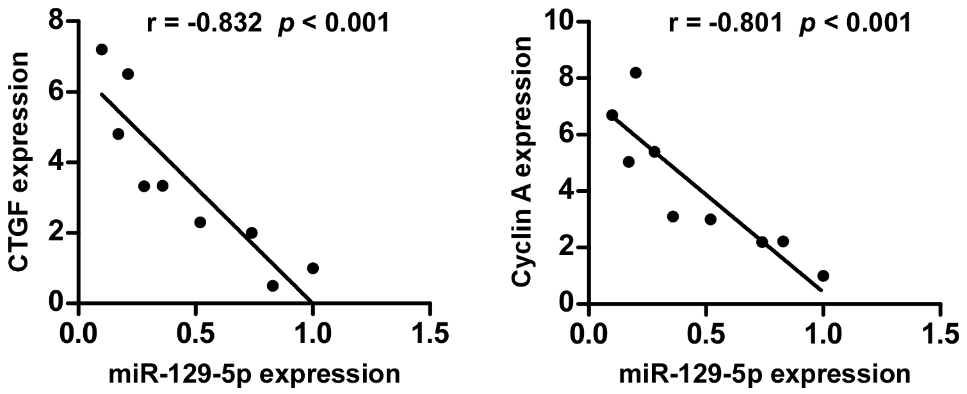

Supplementary Figure 5: Correlation of miR-129-5p expression and *CTGF*, *Cyclin A* mRNA levels in 9 freshly collected human ovarian cancer tissue samples (T).

Supplementary Table 1: Clinicopathological characteristics of studied patients and expression of miR-129-5p in ovarian cancer

Factor	NO.	(%)
**Age (years)**		
≥ 50	96	58.5
< 50	68	41.5
**FIGO Stage**		
I–II	65	39.6
III–IV	99	60.4
**Histological differentiation**		
Grade 1/2	106	64.6
Grade 3	58	35.4
**Pelvic Metastasis**		
No	136	82.9
Yes	28	17.1
**Expression of miR-129-5p**		
Low expression	82	50.0
High expression	82	50.0

Supplementary Table 2: Correlation between the clinicopathological features and expression of miR-129-5p

Patient characteristics		miR-129-5p expression Low	High	*p*-value
**Age (years)**	≥ 50	52	44	0.468
	< 50	30	38	
**FIGO Stage**	I–II	21204	440	0.004
	III–IV	61	38	
**Histological differentiation**	Grade 1/2	37174	69	< 0.001
	Grade 3	45	13	
**Pelvic**	No	59	77	0.006
**Metastasis**	Yes	23	5	

Supplementary Table 3: Univariate and multivariate analysis of different prognostic parameters in patients with gastric cancer by Cox-regression analysis

	Univariate analysis		Multivariate analysis	
	p	**Hazard ratio (95% CI)**	***p***	**Hazard ratio (95% CI)**
**FIGO Stage**				
I–II<br>III–IV	< 0.001	2.315<br>(1.923–2.752)	0.002	2.052<br>(1.430–2.915)
**Histological differentiation**				
Grade 1/2<br>Grade 3	0.042	1.413<br>(1.025–1.936)	0.072	1.936<br>(1.065–2.548)
**Pelvic Metastasis**				
No<br>Yes	0.026	2.214<br>(1.621–2.927)	0.031	2.015<br>(1.238–2.854)
**miR-129-5p expression**				
Low expression<br>High expression	< 0.001	2.025<br>(1.752–2.692)	< 0.001	2.365<br>(1.831–2.952)
